# The Use of Near-Infrared Spectroscopy and/or Transcranial Doppler as Non-Invasive Markers of Cerebral Perfusion in Adult Sepsis Patients With Delirium: A Systematic Review

**DOI:** 10.1177/0885066621997090

**Published:** 2021-03-09

**Authors:** Michael D. Wood, J. Gordon Boyd, Nicole Wood, James Frank, Timothy D. Girard, Amanda Ross-White, Akash Chopra, Denise Foster, Donald. E. G. Griesdale

**Affiliations:** 1 Department of Anesthesiology, Pharmacology and Therapeutics, 8166 University of British Columbia, Vancouver, British Columbia, Canada; 2 Department of Critical Care Medicine, 4257 Queen’s University, Kingston, Ontario, Canada; 3 Department of Physics, 8430 University of Waterloo, Waterloo, Ontario, Canada; 4 Department of Physics, 7497 Brock University, St. Catharines, Ontario, Canada; 5 Department of Critical Care Medicine, University of Pittsburgh, Pittsburgh, PA, USA; 6 Library Services, Queen’s University, Kingston, Ontario, Canada; 7 Department of Medicine, University of British Columbia, Vancouver, British Columbia, Canada; 8 Division of Critical Care Medicine, Department of Medicine, 8166 University of British Columbia, Vancouver, British Columbia, Canada; 9 Center for Clinical Epidemiology & Evaluation, Vancouver Coastal Health Research Institute, Vancouver, British Columbia, Canada

**Keywords:** sepsis, delirium, near-infrared spectroscopy, transcranial Doppler, cerebral autoregulation, pulsatility index

## Abstract

**Background:**

Several studies have previously reported the presence of altered cerebral perfusion during sepsis. However, the role of non-invasive neuromonitoring, and the impact of altered cerebral perfusion, in sepsis patients with delirium remains unclear.

**Methods:**

We performed a systematic review of studies that used near-infrared spectroscopy (NIRS) and/or transcranial Doppler (TCD) to assess adults (≥18 years) with sepsis and delirium. From study inception to July 28, 2020, we searched the following databases: Ovid MedLine, Embase, Cochrane Library, and Web of Science.

**Results:**

Of 1546 articles identified, 10 met our inclusion criteria. Although NIRS-derived regional cerebral oxygenation was consistently lower, this difference was only statistically significant in one study. TCD-derived cerebral blood flow velocity was inconsistent across studies. Importantly, both impaired cerebral autoregulation during sepsis and increased cerebrovascular resistance were associated with delirium during sepsis. However, the heterogeneity in NIRS and TCD devices, duration of recording (from 10 seconds to 72 hours), and delirium assessment methods (e.g., electronic medical records, confusion assessment method for the intensive care unit), precluded meta-analysis.

**Conclusion:**

The available literature demonstrates that cerebral perfusion disturbances may be associated with delirium in sepsis. However, future investigations will require consistent definitions of delirium, delirium assessment training, harmonized NIRS and TCD assessments (e.g., consistent measurement site and length of recording), as well as the quantification of secondary and tertiary variables (i.e., Cox, Mxa, MAP_OPT_), in order to fully assess the relationship between cerebral perfusion and delirium in patients with sepsis.

## Background

Sepsis, which occurs when the body’s response to infection results in the dysfunction of one or more organ systems, is a frequent condition in the intensive care unit (ICU) with approximately 30% of all ICU patients either being admitted for or developing sepsis.^1^ Furthermore, sepsis is the most common cause of death in hospital.^2^ Delirium, a neuropsychiatric syndrome characterized by fluctuating changes in mental status, inattention, altered levels of consciousness, and/or disorganized thinking,^3^ affects up to 80% of ICU patients^4^ and is believed to share many similar pathophysiological mechanisms with sepsis (e.g., inflammation, microvascular damage, and impaired oxidative metabolism).^4–8^ Recently, cerebral oxygenation has also been shown to be significantly lower in septic shock patients with delirium,^9^ suggesting that poor cerebral perfusion may contribute to the delirium development. Furthermore, delirium has been associated with dysfunctional cerebral autoregulation,^10^ which may suggest that secondary neurological injury occurs. Until recently, it has not been feasible to non-invasively quantify surrogate markers of cerebral perfusion in ICU patients.

Near-infrared spectroscopy (NIRS) and transcranial Doppler ultrasonography (TCD) non-invasively measure regional cerebral oxygenation (rSO_2_) and cerebral blood flow (CBF) velocity (CBFV) as proxies of cerebral perfusion, respectively. In NIRS, a sensor and light source are placed on the forehead, where they emit and receive varying wavelengths of near-infrared light (700-1000 nm),^11^ representing changes in oxygenated- and deoxygenated hemoglobin.^12^ In TCD, probes emit an ultrasound wave, which is reflected by red blood cells; the ratio of the frequency of the original and reflected signal is directly proportional to CBFV.^13^ Importantly, NIRS and TCD can be used to quantify additional metrics of cerebral perfusion. For example, cerebral autoregulation can be calculated using a moving Pearson correlation coefficient between mean arterial pressure (MAP) and either rSO_2_ or CBFV, termed the cerebral oximetry index (COx)^14^ and mean flow index (Mxa),^14^ respectively. TCD can be used to determine the pulsatility index (PI), which is the difference between systolic and diastolic CBFV divided by the mean CBFV,^15^ and represents a secondary metric of resistance of the distal cerebral vasculature (reviewed by White and Venkatesh^16^). In addition, the COx and Mxa can be used to identify and maintain MAP thresholds within individualized zones of autoregulation, termed the optimal MAP (MAP_OPT_).^17^ As neurons are dependent on CBF for oxygen delivery and are especially sensitive to hypoxic and ischemic events,^18^ maintaining MAP within patient-specific zones of autoregulation may be crucial to preserving neurological function.

In this systematic review, we aimed to review the clinical literature on the utilization of NIRS and/or TCD as non-invasive surrogates of cerebral perfusion in ICU sepsis patients with and without delirium. The primary objective was to determine whether rSO_2_ and CBFV are associated with delirium during sepsis. As a corollary, we aimed to report NIRS and TCD secondary/tertiary derived variables (e.g., cerebral autoregulation, MAP_OPT_, PI) to investigate potential processes associated with poor cerebral perfusion during delirium. We hypothesize that delirium is the acute manifestation of infection-induced microvascular damage during sepsis, which results in inadequate CBFV and cerebral oxygenation.

## Methods

This systematic review was performed according to the Preferred Reporting Items for Systematic Reviews and Meta-Analyses (PRISMA) statement^19^ (Supplemental Appendix A). Our search was initially conducted during the week of April 04, 2020 in collaboration with content experts and a clinical research librarian who has expertise in systematic reviews (A. R.-W.). We searched Ovid MedLine, Embase (Ovid platform), Cochrane Library (Wiley platform), and Web of Science using a comprehensive combination of subject headings and key words (see Supplemental Appendix B for the complete list of search terms). Relevant articles, as well as those discovered throughout the review process (e.g., searching bibliographies from selected articles for additional resources), were incorporated into the current manuscript. We exported all identified references into a web-based systematic review software platform known as Covidence (Veritas Health Innovation, Melbourne, Australia; www.covidence.org), which used automated algorithms to conduct article deduplication. Using Covidence, 2 of the authors (N.W., and J.F.) independently reviewed each title and abstract to identify articles relevant for full-text review. In the case of disagreement, a third reviewer (M. D. W., or J. G. B.) resolved the conflict. Inclusion criteria were the following: studies using NIRS and/or TCD to measure rSO_2_ and CBFV in critically ill adults (age ≥ 18) with sepsis-associated delirium, sepsis-associated brain dysfunction, or sepsis-associated encephalopathy. We excluded conference proceedings, abstracts, articles published in languages other than English, and those describing animal and human experimental models of sepsis (e.g., lipopolysaccharide injection), primary central nervous system infections, and non-cerebral based NIRS recordings. We did not require specific study designs, types of interventions, and/or delirium assessment methods. We assessed the quality of selected articles using the Modified Newcastle-Ottawa Quality Assessment Form for observational and case-control studies,^20^ as well as the National Institutes of Health Quality Assessment Tool for Case Series (https://www.nhlbi.nih.gov/health-topics/study-quality-assessment-tools). Additionally, we detailed methodologic differences across studies to inform future research design.

Relevant data were extracted from each fully reviewed article using Covidence, which were then exported to Excel (Supplemental File 1). Since many different neuromonitoring devices were identified, coupled with various sensors at several recording periods and durations, as well as a variety of delirium screening methods, we did not synthesize the rSO_2_ and TCD findings with delirium data in a meta-analysis. However, we did contact all primary authors of fully reviewed manuscripts, inviting them to provide either raw or summarized data, which we used to reproduce and further assess associations between poor cerebral perfusion and delirium. If delirium data were originally collected but not reported as the primary outcome, we recoded study data to further assess the association between poor cerebral perfusion and delirium. We used *t*-tests or Mann-Whitney U tests (also known as Wilcoxon rank sum tests) to analyze data that were normally or non-normally distributed, respectively; we considered *P* < .05 to be statistically significant. All figure creation and statistical analyses were performed using R Software (R Foundation for Statistical Computing, Vienna, Austria) Version 3.6.2.

Recently, consensus-based position statements were conducted to update the nomenclature of delirium, encephalopathy, and related terms in the acute care setting to provide a uniform nomenclature.^21^ This important work was further expanded upon to integrate delirium and acute encephalopathy within a single conceptual model (i.e., delirium disorder).^22^ Briefly, the delirium disorder model aims to integrate both the patient and their pathology, while addressing the specific neurophysiology mediating their condition.^22^ As such, this model emphasizes the importance of neurophysiologic disturbances, which mediate the underlying cause of delirium, and assert that there are physiologically distinct delirium disorders that are based on specific acute encephalopathies defined by both etiology (e.g., infection) and neurophysiologic findings (e.g., impaired cerebral perfusion).^22^ Therefore, for the remainder of the review, commonly used terms for acute neurological dysfunction during sepsis (i.e., encephalopathy, brain dysfunction, delirium) will be considered interchangeable for review purposes and will only be referred to as delirium for concision.

## Results

### Study Identification and Inclusion

**Figure 1.**
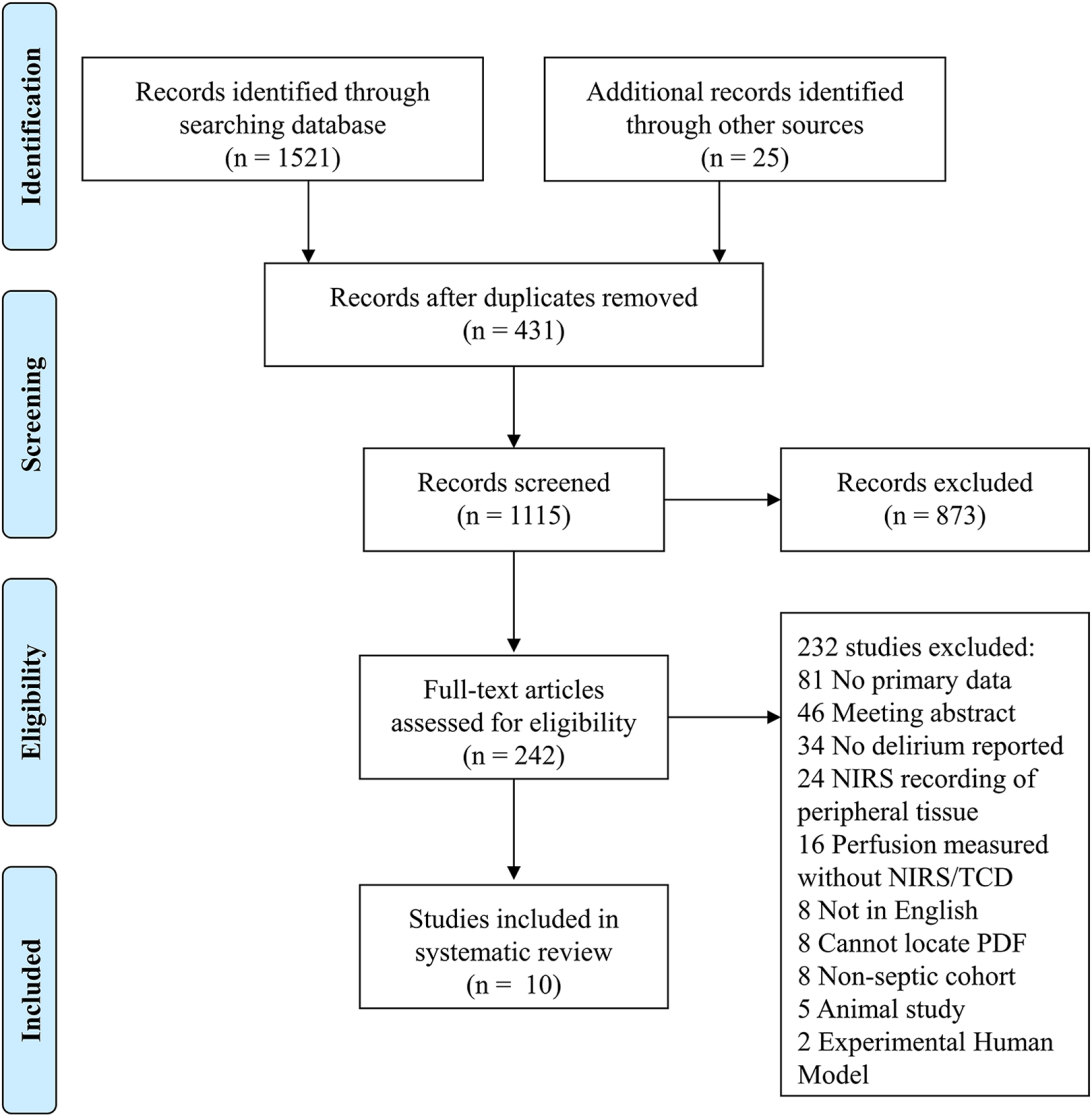
Preferred reporting items for systematic reviews and meta-analyses (PRISMA) flow diagram showing the identification, screening, and eligibility processes, as well as full texts selected for review.

We identified 1115 studies after removing duplicate references (Figure 1). After title and abstract screening, 873 studies were excluded due to relevance (e.g., non-adult, non-septic patients), leaving 242 studies for full-text review. Of these, we excluded 232 studies with the top 4 reasons being: no primary data (n = 81), scientific meeting abstracts (n = 46), no delirium reported/assessed (n = 34), and NIRS recordings of only peripheral (e.g., thenar) tissue (n = 24). Therefore, we included a total of 10 studies after full-text review and 9/10 (90%) of contacted primary authors supplied raw or summarized data. All included studies were conducted in high-income countries (Belgium, Hungary, Canada, Switzerland, USA, and Germany).

### Study Information and Quality

Of the 10 studies selected for review, 6 were prospective cohort studies, 3 were case-control studies, and 1 was a case series (Table 1). Most were recently published, with only one study conducted prior to 2010; 9 studies had a single-center design with only 1 multicenter study being included. The total number of patients included in all 10 studies collectively was 260; sample sizes ranged from 6 to 100. However, only 3 (30%) of these studies reported an a priori sample size calculation to estimate the number of subjects needed for adequate statistical power. All studies included cohorts of patients with sepsis, severe sepsis, and/or septic shock; all studies assessed patients for delirium.

**Table 1.** Study Information and Patient Cohorts.

Study information							
Author	Country	Study population	Setting	Study design	A priori power/sample size calculation	Main outcome	Primary findings
Crippa et al 2018 (n = 100)	Spain & Belgium	All patients treated for sepsis	Multiple ICUs	Prospective cohort	No	Evaluate association of altered cerebral autoregulation with the occurrence of delirium.	Altered cerebral autoregulation was common during sepsis and was an independent predictor of delirium.
Fulesdi et al 2012 (n = 16)	Hungary	Severe sepsis	Single center ICU	Case-control	No	Test whether cerebral vasoreactivity to acetazolamide is impaired in severe sepsis.	Cerebrovascular reactivity is not impaired in patients with severe sepsis.
Funk et al 2016 (n = 15)	Canada	Septic shock	Single center ICU	Prospective cohort	Yes	Determine if the incidence and magnitude of cerebral desaturations is correlated with delirium.	No relationship between the incidence or magnitude of decreases in rSO_2_ were observed with delirium.
Pfister et al 2008 (n = 16)	Switzerland	Sepsis, severe sepsis, or septic shock	Single center ICU	Prospective cohort	No	Assess the association between sepsis-associated delirium and alterations in cerebral perfusion.	Absolute TCD and NIRS values did not differ between patients with and without delirium. However, delirium was associated with dysfunctional cerebral autoregulation.
Pierrakos et al 2014 (n = 38)	Belgium	Sepsis, septic shock	Single center ICU	Prospective cohort	Yes	Assess association between delirium with changes in cerebral vascular resistance.	PI measured within the first 24 hours is associated with the development of delirium.
Rosenblatt et al 2019 (n = 6)	USA	Sepsis, septic shock	Single center ICU	Case series	No	Use continuous autoregulation monitoring using NIRS to identify optimal blood pressure in patients with delirium.	Disturbed autoregulation is associated with increased severity of delirium. MAP_OPT_ varied between patients and over time.
Schramm et al 2012 (n = 30)	Germany	Severe sepsis, septic shock	Single center ICU	Prospective cohort	Yes	Investigated the association between the incidence of delirium and cerebral autoregulatory capacity during sepsis.	Cerebral autoregulation is impaired in the majority of patients, with impairment on day 1 being associated with subsequent development of delirium on day 4.
Szatmari et al 2010 (n = 14)	Hungary	Sepsis-related encephalopathy	Single center ICU	Case-control	No	Test whether acetazolamide induced cerebral vasomotor reactivity is altered in patients with delirium.	Cerebrovascular reactivity is impaired in patients with delirium.
Vasko et al 2014 (n = 15)	Hungary	Severe sepsis	Single center ICU	Case-control	No	Assess rSO_2_ differences among septic patients and controls following acetazolamide administration.	Cerebral vasoreactivity is preserved in patients with delirium.
Wood et al 2016 (n = 10)	Canada	Septic shock	Single center ICU	Prospective cohort	No	Feasibility of NIRS monitoring in septic shock.	NIRS monitoring is feasible and rSO_2_ levels were significantly lower in patients who were delirious for the majority of their ICU stay.

Abbreviations: ICU, intensive care unit; MAP_OPT_, optimal mean arterial pressure; NIRS, near-infrared spectroscopy; PI, pulsatility index; rSO_2_, regional cerebral oxygenation; TCD, transcranial Doppler.

The majority of studies were graded as “good” (n = 8), followed by “fair” (n = 1) and “poor” (n = 1) quality. Specifically, 8/10 (80%) of the included studies had a high-quality patient selection process, 100% of all included studies had adequate follow-up (i.e., low attrition rates) with selected patients, and 5/6 (83%) of the cohort studies used a standardized scale to assess delirium (i.e., CAM-ICU). However, only 2/10 (20%) of all included studies had a high degree of comparability (i.e., statistical adjustment was lacking or not clearly indicated in the majority of studies), and 2/3 (67%) of the case-control studies did not adequately describe how their control populations were selected. See Supplemental Appendix C for complete details. Where available, the demographics and clinical characteristics of each cohort studied were summarized in Table 2.

**Table 2.** Cohort Demographics and Clinical Characteristics.

Study	Age	Sex (% male)	SOFA score	APACHE score	ICU LOS	Mortality, %	Mechanically ventilated, %	Vasoactive agents, %
Crippa et al 2018 (n = 100)	63 (IQR 52-72)	72		21 (IQR 15-26)	7 (IQR 4-13)	24	61	74
Fulesdi et al 2012 (n = 16)	70 (SD 13.7)	56					88	
Funk et al 2016 (n = 15)	57 (SD 14)	40	15 (IQR 12-19)	23 (IQR 18-26)		27	87	100
Pfister et al 2008 (n = 16)	75 (R 18-90)	62		23 (R 9-36)		38	44	44
Pierrakos et al 2014 (n = 38)	66.34 (SD 15.38)	58		21 (SD 6)	25 (SD 11)	63	42	
Rosenblatt et al 2019 (n = 6)	70 (IQR 54-76)	67		34 (R 31-37)	7 (IQR 4-11)	33	50	100
Schramm et al 2012 (n = 30)	64 (SD 17)	67		32 (SD 6)		30	100	100
Szatmari et al 2010 (n = 14)							0	0
Vasko et al 2014 (n = 15)	71 (SD 10.47)	53						
Wood et al 2016 (n = 10)	71 (R 43-85)	40			8.9 (R 2-30)	50	100	100

Abbreviations: SOFA, Sequential Organ Failure Assessment; APACHE, Acute Physiologic Assessment and Chronic Health Evaluation; ICU LOS, intensive care unit length of stay; IQR, interquartile range; R, range.

### Delirium Screening Methods

Delirium was assessed primarily using the Confusion Assessment Method (CAM)-ICU (n = 5), followed by the neurological examination (n = 3), the Glasgow Coma Scale (GCS) score <15 or when disorientation, altered thinking or agitation was reported by the attending physician independent of the use of sedatives/analgesics and in the absence of previous neurological diseases (n = 1), and electronic medical records (n = 1). Importantly, only 1 study clearly indicated that delirium screening was implemented by trained personnel. Despite delirium being an acute fluctuating change in mental status, inattention, altered levels of consciousness, or disorganized thinking,^3^ several of the studies assessed delirium once daily (n = 4), did not clearly indicate the amount of screening (n = 4), and only 2 studies indicated multiple screenings throughout neuromonitoring (n = 2).

### Near-Infrared Spectroscopy-Derived Measures of Regional Cerebral Oxygenation

To quantify rSO_2_, several different devices, sensors, and duration of monitoring were implemented (Table 3). For example, 2 studies used the FORESIGHT (Edwards Lifesciences, USA), 2 studies used the INVOS 5100C (Medtronic, Ireland), and 1 study used the NIRO-200 (HAMAMATSU, Japan). The length of the sensor is directly proportional to the depth at which the signal will penetrate into cerebral tissue;^23^ however, only 1/5 (20%) studies indicated the size of the sensor used. Additionally, 4/5 (80%) studies used bilateral frontotemporal positioning of sensors on the forehead to avoid the superior sagittal sinus, whereas 1 study used one sensor on the middle of the forehead. Furthermore, duration of recording indicated substantial variability. For example, 4/5 (80%) studies used continuous recordings, reported as a mean/median, but the recording length indicated substantial heterogeneity (range 1 to 72 hours); in contrast, 1/5 (20%) studies used discrete recordings (i.e., baseline, 5, 10, 15, and 20 minutes after intervention). Only one study used the NIRS-derived rSO_2_ to further quantify cerebral autoregulation (i.e., COx) and individualized blood pressure (i.e., MAP_OPT_).

**Table 3.** Near-Infrared Spectroscopy and Transcranial Doppler Devices Methodologies.

NIRS monitoring					
Reference	Device	Sensor size	Sensor location	# of sensors	Duration
Funk et al	FORESIGHT	Not specified	Bilateral, forehead	2	Continuous for average 39.8 hours (± 20.4).
Pfister et al	NIRO-200	Not specified	Bilateral, forehead	2	Continuous for 1 hour, reported as median
Rosenblatt et al	INVOS 5100C	Not specified	Bilateral, forehead	2	Continuous for up to 12 hours
Vasko et al	INVOS 5100C	Not specified	Bilateral, forehead	2	Discrete at 0, 5 minutes, 10 minutes, 15 minutes, 20 minutes
Wood et al	FORESIGHT	5 cm	Middle, forehead	1	Continuous for 72 hours, reported as mean
TCD Monitoring					
Reference	Device	Sensor	Sensor Location	# of sensors	Duration
Crippa et al	Doppler-BoxX	2-MHz	Left transtemporal window to insonate the MCA	1	Continuous for 13 (10-18) minutes per patient
Fulesdi et al	Digi-lite	2-MHz	Transtemporal window to insonate the MCA	2	Discrete at 0, 5, 10, 15, 20 minutes after intervention
Pfister et al	Multidop T	2-MHz	Transtemporal window to insonate the MCA	2	Continuous, 1 hour
Pierrakos et al	Not reported	3-MHz	Transtemporal window to insonate the MCA	2	Continuous, 10 seconds
Schramm et al	Doppler BoxX	2-MHz	Transtemporal window to insonate the MCA	2	Continuous, 1-hour daily recordings for 4 consecutive days
Szatmari et al	Digi-Lite	2-MHz	Transtemporal window to insonate the MCA	2	Discrete at 0, 5, 10, 15, 20 minutes after intervention

*Note*. MCA: Middle Cerebral Artery.

### Transcranial Doppler-Derived Measures of Cerebral Blood Flow Velocity

To quantify CBFV, several different devices, sensors, and duration of monitoring were implemented (Table 3). 2 studies used the Digi-Lite (Rimed, Israel), 2 used the Doppler BoxX (Compumedics DWL, Germany), 1 study used the Multidop T (Compumedics DWL, Germany), and 1 study did not report the TCD device used throughout the study. In addition, 5/6 (83%) studies used 2-MHz probes, whereas 1/6 (17%) used a 3-MHz probe. 5/6 (83%) used bilateral placement over the transtemporal window to isonate the middle cerebral artery, where as 1/6 (17%) used only 1 sensor over the left transtemporal window. As with the NIRS monitoring periods mentioned previously, TCD duration of monitoring substantially varied. For instance, 4/6 (67%) studies used continuous monitoring that ranged from 10 seconds to 1 hour, which was reported as a median/mean. Interestingly, one of these studies reported 1-hour daily monitoring for 4 days in the ICU. In contrast, 2/6 (33%) studies reported discrete recordings (i.e., baseline, 5, 10, 15, and 20 minutes after intervention). In regard to reporting a cerebral perfusion/oxygenation index, 3/6 (50%) studies quantified the Mxa, and 3/6 (50%) reported the PI.

### Near-Infrared Spectroscopy and Sepsis-Associated Delirium

**Figure 2.**
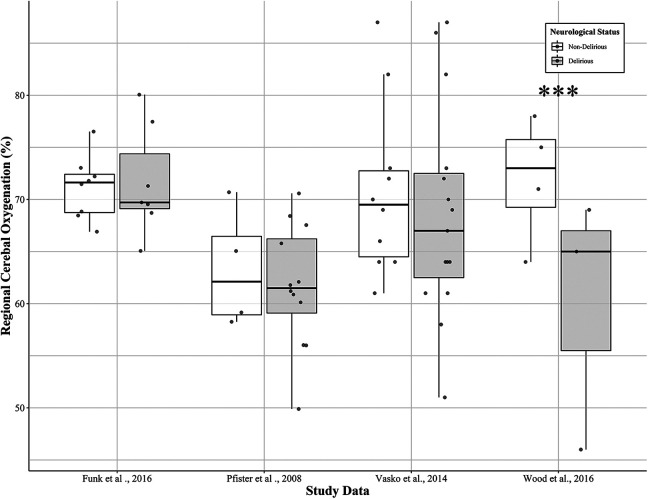
The association between near-infrared spectroscopy (NIRS) derived regional cerebral oxygenation (rSO_2_) and delirium during sepsis. The box represents the interquartile range (IQR), with the black line inside the box representing the median. The whiskers above and below represent the upper quartile +1.5 x IQR or the lower quartile -1.5x IQR, respectively. The dark grey circles represent individual patient rSO_2_ recordings. *Note*. The *** value indicates a significant *t*-test (*p* < 0.001).

As previously stated, several study metrics were reported to assess the association between delirium and poor cerebral perfusion. For example, Funk et al^24^ quantified the percentage of monitoring time below an rSO_2_ of 65%, as well as the area under threshold of 65%, which indicated that there were no significant differences between septic patients with delirium and those with no delirium (*P* > 0.05). Rather than investigating a specific threshold, 2 studies examined^9,25^ if absolute differences in rSO_2_ were significantly different across delirious and non-delirious septic patients. Pfister et al^25^ indicated that although delirious patients had a lower median rSO_2_ (59%, range 49-74) relative to non-delirious patients (65%, range 59-69), it was not statistically significant (*P* = 0.2). In contrast, Wood et al^9^ illustrated that delirious patients had significantly lower rSO_2_ relative to non-delirious patients (64.5% and 72.0%, *P* < .001). To further explore this association, we acquired summary rSO_2_ data from study authors.^9,24–26^ The results are summarized in Figure 2, which indicate that although patients with delirium consistently have a lower absolute median rSO_2_ value across groups, only Wood et al^9^ found a significant difference.

Furthermore, Vasko et al^26^ investigated an absolute change in rSO_2_ after acetazolamide (i.e., vasodilatory stimulus) administration over time (i.e., baseline, 5, 10,15, and 20 minutes after intervention) in both septic patients with delirium and control subjects. This analysis indicated that cerebral vasoreactivity was preserved in delirious patients, as similar increases in rSO_2_ were observed among patients with delirium and controls (8.9 ± 6.5% vs 9.2 ± 4.6%), respectively. Recently, Rosenblatt et al^27^ used continuous rSO_2_ monitoring to identify cerebral autoregulation (i.e., COx) capacity and individual mean arterial pressure (i.e., MAP_OPT_) thresholds in patients with mild (GCS ≥13) or moderate-to-severe (GCS < 13) delerium. Both absolute rSO_2_ (52.32% vs 48.94%, *P* > 0.05) and MAP_OPT_ (83 vs 85, *P* > 0.05) were not significantly different among the mild and moderate-severe delirium, respectively. In contrast, COx values were significantly higher (*P* < 0.001) in patients with moderate to severe delirium (0.19 ± 0.13) compared with patients with mild delirium (0.00 ± 0.13) indicating dysfunctional autoregulation with increasing delirium severity. See Table 4 for further detail across studies.

**Table 4.** NIRS Neuromonitoring Differences Between Neurological Outcomes.

	Clinical outcomes		NIRS						Delirium screening
Study	Subgroup	Sample size	rSO_2_ Metrics	*P* value	COx	*P* value	MAP_OPT_	*P* value	Method
Funk et al 2016	Delirium	7	71.69 (SD 5.24)	>.05					CAM-ICU
	No delirium	8	71.15 (SD 3.04)						
Pfister et al 2008	Delirium	12	59% (IQR 49-74)	>.05					CAM-ICU
	No delirium	4	65% (59-69)						
Rosenblatt et al 2019	Mild delirium (GCS ≥ 13)*	3	48.94 (SD 7.32)	>.05	0.19 ± 0.13	<.001	85.00 (SD 13.23)	>.05	EMR indicating altered mental status (e.g., inattention)
	Moderate-severe delirium (GCS < 13) *	3	52.32 (SD 13.72)		0.00 ± 0.13		83.33 (SD 16.07)		
Vasko et al 2014	Delirium	15	70.80 (SD 8.23)	> .05					Neurologic exam
	Controls	20	68.60 (SD 10.20)						
Wood et al 2016	Delirium for majority of admission	3	60 (SD 12.29)	<.0001					CAM-ICU
	No delirium for majority of admission	4	72 (SD 6.06)						

Abbreviations: CAM-ICU, Confusion Assessment Method for the intensive care unit; COx, cerebral oximetry index; EMR, electronic medical record; GCS, Glasgow Coma Scale; IQR, interquartile range; NIRS, near-infrared spectroscopy; rSO_2_, regional cerebral oxygenation.

* GCS: Glasgow Coma Scale. Severity of delirium was indicated GCS (i.e., ≥13 to indicate mild delirium, whereas GCS <13 indicated moderate-to-severe delirium).

### Transcranial Doppler and Sepsis-Associated Delirium

**Figure 3.**
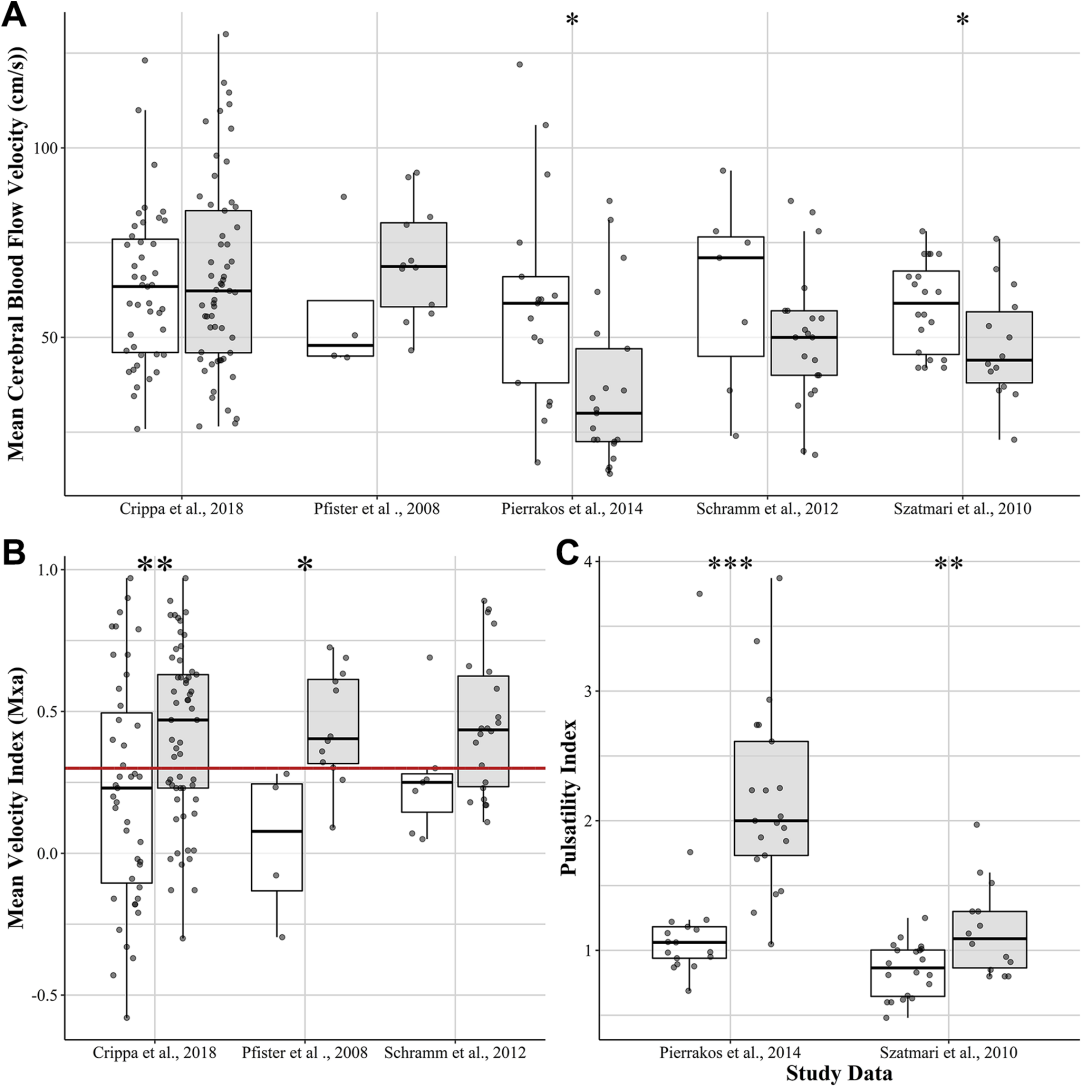
Transcranial Doppler (TCD) derived metrics of cerebral perfusion and their association with delirium. (**A)** The majority of reviewed studies indicate that mean cerebral blood flow velocity is lower in delirious septic patients. (**B)** Delirious patients consistently indicate impaired cerebral autoregulation. *Note.* The red line indicates the commonly used 0.3 mean velocity index cut-off to denote dysfunctional cerebral autoregulation. (**C)** Pulsatility index is consistently higher among delirious patients indicating potential reduced vasomotor reactivity. The whiskers above and below represent the upper quartile +1.5 x IQR or the lower quartile -1.5x IQR, respectively. The dark gray circles represent individual patient rSO_2_ recordings. *Note*. *P < .05; **P < .01; ***P < .001.

Using absolute values of CBFV generated using TCD, there appears to be marked heterogeneity across studies when assessing differences between septic patients with and without delirium. For example, 3 studies indicated a non-significant difference between groups,^25,28–30^ whereas the 2 remaining studies demonstrated that patients with delirium had significantly lower CBFV relative to non-delirious patients^31^ and controls.^32^ These results are summarized in Table 5, and when data were provided by authors, in Figure 3 A. Using CBFV, 3 studies further quantified cerebral autoregulatory capacity (i.e., Mxa) during delirium^25,28,29^ (Figure 3 B), which indicated that delirious patients consistently had higher median Mxa values (i.e., dysfunctional autoregulation). Specifically, Crippa et al^28^ and Pfister et al^25^ reported that delirious patients had significantly higher Mxa values relative to non-delirious septic patients (0.47 and 0.23, *P* < 0.01; 0.40 and 0.08, *P* < .05), respectively. Similar to the 3 of 4 NIRS studies with absolute rSO_2_, both TCD studies did not observe a significant difference in absolute CBFV values. Schramm et al^29^ indicated that although delirious patients had a higher average Mxa value (0.44, IQR 0.24-0.63) during the first day of sepsis, this was not significantly higher than non-delirious septic patients (0.25, IQR 0.15-0.28).

**Table 5.** TCD Neuromonitoring Differences Between Neurological Outcomes.

	Clinical outcomes			TCD metrics					Delirium screening
Study	Subgroup	Sample size	Mean flow velocity(cm/s)	*P* value	Pulsatility index*	*P* value	Mean flow index (Mxa)	*P* value	Screening tool
Crippa et al 2018	Delirium	57	66 ± 25.1	>.05			0.47 (IQR 0.21-0.64)	<.01	GCS < 15 or altered mental status reported by physician
	No Delirium	43	63.1 ± 20.3				0.23 (IQR -0.12 – (0.52)		
Fulesdi et al 2012	Delirious	16	52.9 ± 29.4	>.05	1.16 ± 0.24	<.001			Neurological examination
	Control	16	56.6 ± 10.7		0.84 ± 0.21				
Pfister et al 2008	Delirium	12	76 (R 40–97)	>.05			0.40 (0.32-0.61)	<.05	CAM-ICU
	No Delirium	4	48 (R 45–98)				0.08 (-0.13 – 0.24)		
Pierrakos et al 2014	Delirium	21	36.56 ± 21.77	<.05					CAM-ICU
	No Delirium	17	59.06 ± 27.85						
Schramm et al 2012	Delirium	22	49.90 ± 17.83	>.05	2.16 ± 0.69	<.001	0.44 (IQR 0.24-0.63)	>.05	CAM-ICU
	No Delirium	7	61.71 ± 24.88		1.22 ± 0.69		0.25 (IQR 0.15 - 0.28)		
Szatmari et al 2010	Delirious	14	47.9 ± 14.5	<.05	1.15 ± 0.35,	<.01			Neurological examination
	Control	20	58.2 ± 12.0		0.85 ± 0.2				

Abbreviations: CAM-ICU, Confusion Assessment Method for the intensive care unit; GCS, Glasgow Coma Scale; IQR, interquartile range; MFV, flow velocity; Mxa, mean flow index; R: range; TCD, transcranial Doppler.

*Note*. For Fulesdi et al and Szatmari et al, only baseline values are reported prior to study intervention. Only day 1 values were compared for Schramm et al

Building on this notion of impaired cerebral perfusion, cerebrovascular resistance was further quantified using the PI in 3 studies.^30–32^ When compared with controls and non-delirious patients, patients with delirium consistently displayed higher PI values (see Table 5 for complete details and Figure 3 C for available study data). As all 3 studies indicated that CBFV was lower among patients with delirium and the PI was substantially higher, it provides preliminary evidence of increased cerebrovascular resistance and potentially impaired perfusion. Unfortunately, cerebral autoregulatory capacity was not directly accessed in these patients.

## Discussion

Our previous work explored the association between rSO_2_ and delirium in all critically ill patients, which indicated that delirious patients had lower cerebral oxygenation values.^33^ However, informative secondary-derived variables of cerebral perfusion (e.g., autoregulation) were either not quantified by original study authors or were not reported in our previous review. Furthermore, delirium shares many similar pathophysiological mechanisms with sepsis (e.g., inflammatory cytokines, microvascular damage, impaired oxidative metabolism).^4–8^ As such, patients with sepsis are particularly prone to developing delirium.^4^ Therefore, in the current systematic review, we aimed to critically evaluate and synthesize the clinical literature on the utilization of non-invasive surrogates of cerebral perfusion (i.e., NIRS and/or TCD) in ICU sepsis patients with and without delirium. The primary objective was to assess the relationship between poor rSO_2_ and CBFV with the presence of delirium, as well as report NIRS and TCD secondary/tertiary derived variables to investigate potential processes associated with inadequate cerebral perfusion. Despite an expansive search, only 10 studies have been published, mostly within the last 10 years. However, a wide array of NIRS and TCD neuromonitoring devices were identified with marked heterogeneity among monitoring periods and delirium assessment tools. Though absolute values of the NIRS-derived rSO_2_ signal were consistently lower in patients with delirium than in non-delirious/control subjects, only 1 of these studies found a statistically significant difference. One study further quantified cerebral autoregulatory capacity using COx, which indicated that delirium severity was associated with dysfunctional autoregulation. Using COx, MAP_OPT_ was also calculated across patients, which indicated MAP thresholds substantially higher than the current >65 mmHg ICU guidelines.^34^ Although trends in absolute TCD-derived CBFV values were inconsistent across studies, the 3 studies that also used CBFV to calculate autoregulatory capacity (i.e., Mxa) consistently demonstrated that patients with delirium had impaired cerebral autoregulation. Interestingly, in 3 of the studies a low CBFV was observed concurrently with a high PI, which suggests an increase in cerebrovascular resistance resulting in decreased CBFV. Taken together, our review provides preliminary evidence that poor cerebral perfusion is a potential pathophysiological mechanism associated with delirium development during sepsis, which warrants further investigation.

Although the underlying cause of delirium remains unclear, our review indicates that poor cerebral perfusion is likely an important pathophysiological mechanism underpinning delirium in sepsis, which aligns with other previous sepsis reviews.^35–38^ Our review specifically focused on the use of NIRS and/or TCD as surrogate markers of cerebral perfusion during delirium, as both devices have been shown to correlate with other indices of cerebral perfusion (e.g., jugular venous bulb oxygen saturation^39^ and positron emission tomography derived CBF^40^), and invasive neuromonitoring is not routinely implemented outside of patients with traumatic brain injury. Although the majority of studies included in our review indicated poor cerebral perfusion in patients with delirium, septic patients have also been shown to have increased CBF (potentially indicating cerebral hyperperfusion), as measured using arterial spin labeling magnetic resonance imaging, relative to controls.^41^ However, delirium was not directly assessed in these patients. Using Xenon-enhanced computed tomography in critically ill patients, Yokota et al^42^ demonstrated decreased CBF during hypoactive delirium, which normalized when delirium resolved; it remains to be seen if this finding occurs in sepsis patients with delirium. Additionally, the majority of included studies did not identify if patients were delirious at the time of neuromonitoring or were unclear about the timing of assessments (data not shown). As such, it is unclear if altered cerebral perfusion is a precursor to delirium, occurs during delirium, resolves after delirium cessation, or a combination of all of the above. Taken together, alterations in cerebral perfusion are common in sepsis and further investigation is needed to investigate if these derangements in cerebral hemodynamics occur during delirium and resolve upon delirium cessation. Additional investigations are warranted to assess if delirium subtypes (i.e., hypoactive, hyperactive, mixed) share similar pathophysiological mechanisms, as this was not systematically documented across reviewed studies.

In the few studies that quantified cerebral autoregulatory capacity (i.e., the cerebrovascular reflex mechanism that maintains stable and adequate CBF during fluctuations in MAP^43^) either by NIRS-derived COx or TCD-derived Mxa, septic patients with delirium consistently displayed autoregulatory dysfunction. Given that the NIRS- and TCD-derived measures of cerebral autoregulation have been shown to be positively associated^44,45^ and have good agreement,^14,46^ it is not surprising that this finding was observed across device metrics. Despite quantifying cerebral autoregulation in 4 of the 10 included studies, only one study used these recordings to further calculate MAP_OPT_ thresholds. Previous studies among other critically ill populations (e.g., cardiac arrest,^47,48^ traumatic brain injury^49,50^) have demonstrated that the zone of autoregulation may be narrowed with marked between-patient heterogeneity. Furthermore, improved neurologic outcomes have been associated with higher MAP values in patients post-cardiac arrest^51,52^ and a recent review of acutely ill patients indicated those whom differed substantially from their MAP_OPT_ or optimal cerebral perfusion were more likely to have an unfavorable outcome.^53^ Importantly, Rosenblatt et al^27^ illustrated that MAP_OPT_ was highly variable between patients and within individuals (data not shown) over time, and the median MAP_OPT_ (i.e., 85 mmHg; IQR, 70-100) was substantially greater than 65 mmHg. Cerebral hypo- and hyper-perfusion could result from a MAP below or above the lower and upper limits of autoregulation, respectively. However, the zone of autoregulation was not quantified among delirious patients. Furthermore, a largescale multicenter trial comparing high (80-85 mmHg) vs low (65-70 mmHg) resuscitation MAP targets did not demonstrate a significant difference in mortality or the frequencies of ischemic events between groups.^54^ This study also indicated potential adverse effects (i.e., significantly more episodes of atrial fibrillation) with higher MAP targets in patients with sepsis.^54^ However, this trial did not quantify MAP_OPT._ Therefore, it remains to be seen if quantifying individualized MAP_OPT_ targets, as well as dysfunctional cerebral autoregulation capacity, among sepsis patients will reduce brain injury (e.g., cerebral ischemia) and improve outcomes among patients with sepsis.

Two studies investigated potential mechanisms associated with dysfunctional autoregulation. Schramm et al^29^ indicated that inflammatory markers (e.g., C-reactive protein, procalcitonin) were associated with dysfunctional autoregulation, however, this was only associated with day 2 of monitoring. Pfister et al^25^ further verified that C-reactive protein was associated with dysfunctional autoregulation, however, no association was observed between other inflammatory markers (e.g., interleukin-6) and dysfunctional autoregulation. Therefore, further investigation into the association between dysfunctional cerebral autoregulation and inflammatory biomarkers is warranted to identify mechanistic pathways.

TCD is frequently implemented in neurologic ICUs to detect abnormalities of cerebral hemodynamics. Interestingly, 4/6 (67%) studies that included TCD monitoring indicated that patients with delirium had decreased CBFV (although not statistically significant) relative to non-delirious/control subjects. This decrease in CBFV could be associated with multiple clinical factors such as elevated ICP, inadequate MAP, reduced CBF or cardiac output, reduced partial pressure of carbon dioxide, and/or the use of anesthetics,^55^ but remains unanswered by solely quantifying CBFV. Fortunately, 3/6 (50%) studies calculated the PI. When in tandem, decreased CBFV and a high PI potentially indicate vasoconstriction, and thus increased cerebrovascular resistance. Szatmari et al^32^ indicated that the PI values were higher among delirious patients relative to controls throughout a 20-minute time course after acetazolamide (i.e., vasodilatory stimulus)^56^ administration, and provided evidence that cerebral vasoreactivity was impaired (i.e., occurred slower and was lower in magnitude) during delirium. However, this finding was inconsistent across studies.^30^ As the PI has been positively associated with intracranial pressure and negatively related to cerebral perfusion pressure,^57,58^ an increased PI may also indicate secondary neurological injury. However, none of the reviewed studies that quantified the PI collected a comprehensive assortment of biomarkers to assess the potential underlying pathophysiological mechanisms, as well as assess if this alteration in perfusion was primarily from neurological dysfunction or secondary to a cardiac complication (e.g., ventricular dysfunction, valvular insufficiency), and thus the processes associated with increased cerebrovascular resistance remain unanswered.

The primary limitation of our review is reporting bias; 83 trials were excluded due to no primary data being available, and 8 study manuscripts could not be obtained, which may have biased our review toward studies with only significant outcomes. However, several of the studies that were excluded were systematic reviews and editorials of sepsis, which highlights an important gap in critical care research as delirium was not the primary focus. Another limitation is that included studies were heterogeneous in regard to design and methodology, which limited our ability for data synthesis. Additionally, several of the included studies did not use validated delirium screening and severity assessment tools, such as the CAM-ICU^59^ and CAM-ICU 7,^60^ which may have caused discrepancies across studies regarding neurological status. Moreover, delirium was often assessed once daily throughout the monitoring period and type of delirium (e.g., hypo-, hyper-active, mixed), as well as if patients were delirious at the time of neuromonitoring, was often not clearly or directly stated. Thus, it is still unclear if altered cerebral perfusion is similar across delirium subtypes and if these neurophysiologic alterations resolve concurrently with delirium cessation. Furthermore, as excessive burst suppression has been previously associated with delirium,^61,62^ as well as increased mortality in the ICU,^63^ electroencephalography may be another viable non-invasive neuromonitoring device to provide additional neurological information as an adjunct to NIRS and/or TCD monitoring. Additionally, numerous studies did not report an a priori power analysis and had small sample sizes (n < 20), which may limit the validity of study findings. Importantly, none of the included studies assessed the association between poor cerebral perfusion and long-term patient-oriented outcomes (e.g., cognition, quality of life). Furthermore, we cannot discount the fact that unmeasured or residual cofounding (e.g., due to medications, metabolic abnormalities, hemodynamic instability, altered neurotransmission) may have biased study findings. Although our review has identified that sepsis patients with delirium may have altered cerebral perfusion, there were minimal data provided to assess potential mechanisms associated with this dysfunction and further work is needed to directly investigate if altered cerebral perfusion is the result of primary or secondary injury in septic patients with delirium.

## Conclusions

We hypothesized that delirium is the acute manifestation of infection-induced microvascular damage during sepsis, which results in inadequate CBFV and cerebral oxygenation. This systematic review demonstrates that poor cerebral perfusion may be associated with delirium during sepsis. To increase the validity and reliability of study findings, future investigations will require adequate statistical power to model and control for covariates and confounds, implement consistent delirium assessment tools across studies, harmonize NIRS and TCD assessments (e.g., consistent measurement site and length of recording), as well as the quantification of secondary and tertiary variables (i.e., Cox, Mxa, MAP_OPT_), to adequately assess the relationship between cerebral perfusion and delirium in patients with sepsis.

## Supplemental Material

Supplemental Material, sj-pdf-1-jic-10.1177_0885066621997090 - The Use of Near-Infrared Spectroscopy and/or Transcranial Doppler as Non-Invasive Markers of Cerebral Perfusion in Adult Sepsis Patients With Delirium: A Systematic Review Click here for additional data file. Supplemental Material, sj-pdf-1-jic-10.1177_0885066621997090 for The Use of Near-Infrared Spectroscopy and/or Transcranial Doppler as Non-Invasive Markers of Cerebral Perfusion in Adult Sepsis Patients With Delirium: A Systematic Review by Michael D. Wood, J. Gordon Boyd, Nicole Wood, James Frank, Timothy D. Girard, Amanda Ross-White, Akash Chopra, Denise Foster and Donald. E. G. Griesdale in Journal of Intensive Care Medicine

Supplemental Material, sj-pdf-2-jic-10.1177_0885066621997090 - The Use of Near-Infrared Spectroscopy and/or Transcranial Doppler as Non-Invasive Markers of Cerebral Perfusion in Adult Sepsis Patients With Delirium: A Systematic Review Click here for additional data file. Supplemental Material, sj-pdf-2-jic-10.1177_0885066621997090 for The Use of Near-Infrared Spectroscopy and/or Transcranial Doppler as Non-Invasive Markers of Cerebral Perfusion in Adult Sepsis Patients With Delirium: A Systematic Review by Michael D. Wood, J. Gordon Boyd, Nicole Wood, James Frank, Timothy D. Girard, Amanda Ross-White, Akash Chopra, Denise Foster and Donald. E. G. Griesdale in Journal of Intensive Care Medicine

Supplemental Material, sj-pdf-3-jic-10.1177_0885066621997090 - The Use of Near-Infrared Spectroscopy and/or Transcranial Doppler as Non-Invasive Markers of Cerebral Perfusion in Adult Sepsis Patients With Delirium: A Systematic Review Click here for additional data file. Supplemental Material, sj-pdf-3-jic-10.1177_0885066621997090 for The Use of Near-Infrared Spectroscopy and/or Transcranial Doppler as Non-Invasive Markers of Cerebral Perfusion in Adult Sepsis Patients With Delirium: A Systematic Review by Michael D. Wood, J. Gordon Boyd, Nicole Wood, James Frank, Timothy D. Girard, Amanda Ross-White, Akash Chopra, Denise Foster and Donald. E. G. Griesdale in Journal of Intensive Care Medicine
