# Supplementary material for: The Use of Near-Infrared Spectroscopy and/or Transcranial Doppler as Non-Invasive Markers of Cerebral Perfusion in Adult Sepsis Patients With Delirium: A Systematic Review
Source: J Intensive Care Med. 2021 Mar 9;37(3):408–22. doi: 10.1177/0885066621997090 (PMC8772019; doi:10.1177/0885066621997090)
Supplement: Supplemental Material, sj-pdf-2-jic-10.1177_0885066621997090 - The Use of Near-Infrared Spectroscopy and/or Transcranial Doppler as Non-Invasive Markers of Cerebral Perfusion in Adult Sepsis Patients With Delirium: A Systematic Review [file sj-pdf-2-jic-10.1177_0885066621997090.pdf]

Database: Ovid MEDLINE(R), Ovid MEDLINE(R) Daily and Epub Ahead of Print, In-Process & Other Non-Indexed Citations <1946 to Present>
Search Strategy:
----
1 (brain adj3 oximetry).mp. (72)
2 (brain adj3 oxymetry).mp. (8)
3 (cerebral adj3 oximetry).mp. (714)
4 (cerebral adj3 oxymetry).mp. (23)
5 Spectroscopy, Near-Infrared/ (12996)
6 near-infrared spectroscop*.mp. (11609)
7 NIRS.ab,ti. (5733)
8 Ultrasonography, Doppler, Transcranial/ (7320)
9 trans-cranial doppler.mp. (72)
10 transcranial doppler.mp. (8240)
11 cerebral blood velocity.mp. (132)
12 cerebral blood flow.mp. (31544)
13 (cerebral adj3 oxygenat*).mp. (2747)
14 (brain adj3 oxygenat*).mp. (1119)
15 Blood Flow Velocity/ (57283)
16 cerebral autoregulation.mp. (1996)
17 optimal mean arterial pressure*.mp. (18)
18 individuali?ed mean arterial pressure*.mp. (0)
19 MAPOPT.mp. (10)
20 Oxygen Consumption/ (102963)
21 exp Brain/ (1192892)
22 (brain or cerebral).mp. (1640445)
23 (15 or 20) and (21 or 22) (19656)
24 exp Sepsis/ (121370)
25 Sepsis-Associated Encephalopathy/ (77)
26 sepsis.mp. (123091)
27 septic shock.mp. (21948)
28 exp Delirium/ (9231)
29 ((septic or sepsis) adj3 (delirium or encephalopathy)).mp. [mp=title, abstract, original title, name of substance word, subject heading word, floating sub-heading word, keyword heading word, organism supplementary concept word, protocol supplementary concept word, rare disease supplementary concept word, unique identifier, synonyms] (572)
30 or/1-19,23 (118389)
31 or/24-29 (193141)
32 30 and 31 (703)
33 (exp infant/ or exp child/ or adolescent/) not exp adult/ (1857720)
34 32 not 33 (642)
35 animals/ not humans.sh. (4651925)
36 34 not 35 (428)
37 limit 36 to english language (408)

****

1.

In vitro quantification of lactate in Phosphate Buffer Saline (PBS) samples.
Budidha K; Mamouei M; Baishya N; Vadgama P; Kyriacou PA.
Conference Proceedings: ... Annual International Conference of the IEEE Engineering in Medicine & Biology Society. 2019:1205-1208, 2019 07.
[Journal Article. Research Support, Non-U.S. Gov't]
UI: 31946109
Authors Full Name
Budidha, K; Mamouei, M; Baishya, N; Vadgama, P; Kyriacou, P A.
