# Supplementary material for: The Use of Near-Infrared Spectroscopy and/or Transcranial Doppler as Non-Invasive Markers of Cerebral Perfusion in Adult Sepsis Patients With Delirium: A Systematic Review
Source: J Intensive Care Med. 2021 Mar 9;37(3):408–22. doi: 10.1177/0885066621997090 (PMC8772019; doi:10.1177/0885066621997090)
Supplement: Supplemental Material, sj-pdf-3-jic-10.1177_0885066621997090 - The Use of Near-Infrared Spectroscopy and/or Transcranial Doppler as Non-Invasive Markers of Cerebral Perfusion in Adult Sepsis Patients With Delirium: A Systematic Review [file sj-pdf-3-jic-10.1177_0885066621997090.pdf]

Supplemental Appendix C. Quality Assessment Across Cohort, Case-Control, and Case Series Studies.

Modified Newcastle–Ottawa Quality Assessment Scale criteria: Cohort Studies

**Study**	**Selection**				**Comparability**	**Outcome**			
	**Representativeness of Exposed Cohort**	**Selection of the Non-Exposed Cohort from Same Source as Exposed Cohort**	**Ascertainment of Exposure**	**Outcome of Interest Was Not Present at Start of Study**	**Comparability of Cohorts**	**Assessment of Outcome**	**Follow-Up Long Enough for Outcome to Occur**	**Adequacy of Follow-Up**	**Quality Score**
Crippa et al. (2018)	Participants were truly representative of patients treated for sepsis. Sepsis was defined using the 2001 SCCM/ESICM/ACCP/ ATS/SIS International Sepsis Definitions Conference. F	Yes F	Diagnosis of delirium was based on Glasgow Coma Scale score, was not standardized on specific scales or scores related to delirium.	Yes F	A) Clearly controls for co-morbidities and other factors such as age and pregnancy. F<br>B) Binary logistic regression analyses were performed with delirium as the dependent variable. Not clearly stated if confounders were adjusted for.	Neurophysiologic monitoring using transcranial doppler to measure blood flow velocity and mean arterial pressure signals. Clinicians were blinded to the cerebral autoregulation assessments. F	Yes F	94% of patients were accounted for with 6 patients excluded due to artefacts. F	Good
Funk et al. (2016)	Participants were truly representative of patients with severe sepsis and septic shock Definition were based on the American College of Chest Physicians/Society of Critical Care Medicine Consensus Conference Committee. F	No – cerebral desaturations were compared with concurrent trial data (i.e., patients undergoing high-risk non-cardiac surgical procedures).	Delirium was measured by the Confusion Assessment Method for the Intensive Care Unit (CAM-ICU) score. F	Yes F	A) Does not control for co-morbidities or any other factors.<br>B) Between group continuous variables were analyzed with a Student t test or a Mann-Whitney U test, depending on the distribution of the data. Not clearly stated if confounders were adjusted for.	Neurophysiologic monitoring using bi-lateral near-infrared spectroscopy to measure overall number of cerebral desaturations, percent time under threshold, and the area under threshold, as well as the magnitude of the decrease. F	Yes F	Complete follow up – all subjects accounted for. F	Poor
Pfister et al. (2008)	Participants were truly representative of patients with severe sepsis and septic shock. Sepsis was defined using the 2001 SCCM/ESICM/ACCP/ ATS/SIS International Sepsis Definitions Conference. F	Yes F	Delirium was measured by the Confusion Assessment Method for the Intensive Care Unit (CAM-ICU) score. F	Yes F	A) Clearly controls for co-morbidities. F<br>B) Comparisons were made using the Mann-Whitney *U* test. Not clearly stated if confounders were adjusted for.	Multimodal neurophysiologic monitoring using transcranial doppler and near-infrared spectroscopy. F	Yes F	69% of investigated patients were included. Seven patients had to be excluded.	Good

Pierrakos et al. (2014)	Participants were truly representative of patients with sepsis. Sepsis was defined using the 2001 SCCM/ESICM/ACCP/ ATS/SIS International Sepsis Definitions Conference. F	Yes F	Delirium was measured by the Confusion Assessment Method for the Intensive Care Unit (CAM-ICU) score. F	Yes F	A) Clearly controls for co-morbidities and other factors such as age and drug intoxication. F<br>B) Multivariable logistic regression. Not clearly stated if confounders were adjusted for.	Neurophysiologic monitoring using transcranial doppler. The pulsatility index and cerebral blood flow index were derived from blood velocity in the middle cerebral artery. F	Yes F	95% of selected patients were included in the analysis. Unlikely to introduce bias. F	Good
Schramm et al. (2012)	Participants were truly representative of patients with severe sepsis or septic shock. Sepsis was defined using the 2001 SCCM/ESICM/ACCP/ ATS/SIS International Sepsis Definitions Conference. F	Yes	Delirium was measured by the Confusion Assessment Method for the Intensive Care Unit (CAM-ICU) score. F	Yes F	A) Clearly controls for co-morbidities and other factors such as pregnancy. F<br>B) Analysis of variance, correlations between autoregulation, APACHE II scores, age and serum markers were performed. F	Neurophysiologic monitoring using transcranial doppler to measure cerebral blood flow velocity. Invasive arterial blood pressure and haemodynamic monitoring were measured using a PiCCO-Catheter. F	Yes F	97% of selected patients were followed for the entire study. F	Good
Wood et al. (2016)	Participants were truly representative of patients with severe sepsis or septic shock. All patients met the Society of Critical Care Medicine's criteria for either severe sepsis or septic shock. F	Yes F	Delirium was measured by the Confusion Assessment Method for the Intensive Care Unit (CAM-ICU) score. F	Yes F	A) Clearly controls for co-morbidities. F<br>B) T-tests with Bonferroni correction. Not clearly stated if confounders were adjusted for.	Neurophysiologic monitoring using near-infrared spectroscopy to assess cerebral oxygenation for the first 72 hours of admission. F	Yes F	Complete follow up – all subjects accounted for. F	Good

Note. Good quality: 3 or 4 stars (F) in selection domain AND 1 or 2 stars in comparability domain AND 2 or 3 stars in outcome domain; Fair quality: 2 stars in selection domain AND 1 or 2 stars in comparability domain AND 2 or 3 stars in outcome/exposure domain; Poor quality: 0 or 1 star in selection domain OR 0 stars in comparability domain OR 0 or 1 stars in outcome/exposure domain. Comparability: A) Controls for co-morbidities and/or other factors during recruitment B) Controls for confounding factors during statistical analysis.

Modified Newcastle–Ottawa Quality Assessment Scale criteria: Case Control Studies

Study	Selection				Comparability	Exposure			Quality Score
	Case Definition With Independent Validation	Representativeness of the cases	Selection of Controls	Definition of Controls	Comparability of Cases and Controls	Ascertainment of Exposure	Same Method of Ascertainment?	Non-response Rate	

Fulesdi et al. (2012)	Cases met the criteria of severe sepsis according to the American College guidelines. A certified neurologist performed a detailed neurologic assessment to exclude direct infectious involvement of the central nervous system. F	Cases were consecutively enrolled in the study. F	Not identified if hospital or community controls.	Non-septic healthy persons without previous diseases affecting cerebral vasoreactivity. F	A) Clearly controls for co-morbidities and other factors such as age and sex. F<br>B) Repeated measure analysis of variance was implemented. When significant differences were detected, pairwise comparisons were performed between the groups using the Mann-Whitney U test. Not clearly stated if confounders were adjusted for.	Neurophysiologic monitoring using transcranial Doppler to measure blood flow velocities at rest and at 5, 10, 15, and 20 minutes after intravenous administration of 15 mg/kg acetazolamide. Arterial blood was taken from a radial artery catheter in septic patients to perform blood gas analysis. Placement of arterial catheters in healthy control was considered unethical. F	Yes F	Complete follow up – all subjects accounted for. F	Good
Szatmari et al. (2010)	Patients had to meet the criteria of clinical sepsis and had to show disturbance of consciousness or alertness of any severity. A certified neurologist performed a detailed neurological assessment to exclude direct infectious involvement of the central nervous system. F	Not stated if consecutive enrollment of patients but is a representative series of cases. F	Not identified if hospital or community controls.	Non-septic healthy persons without previous diseases affecting cerebral vasoreactivity. F	A) Clearly controls for co-morbidities and other factors such as age and sex. F<br>B) Repeated measure analysis of variance was implemented. When significant differences were detected, pairwise comparisons were performed between the groups using the Mann-Whitney U test. Not clearly stated if confounders were adjusted for.	Neurophysiologic monitoring using transcranial Doppler to measure blood flow velocities at rest and at 5, 10, 15, and 20 minutes after intravenous administration of 15 mg/kg acetazolamide. Arterial blood was taken from a radial artery catheter in septic patients to perform blood gas analysis. Placement of arterial catheters in healthy control was considered unethical. F	Yes F	Complete follow up – all subjects accounted for. F	Good
Vasko et al. (2014)	All patients fulfilled the criteria of severe sepsis according to the guidelines of the American College of Chest Physicians. A certified neurologist performed a detailed neurological assessment to exclude direct infectious involvement of the central nervous system. F	Not stated if consecutive enrollment of patients but is a representative series of cases. F	No description.	Refers to controls as "healthy controls" without defining "healthy".	A) Clearly controls for co-morbidities and other factors such as age and sex. F<br>B) Repeated measure analysis of variance was implemented. When significant differences were detected, pairwise comparisons were performed between the groups using the Mann-Whitney U test. Not clearly stated if confounders were adjusted for.	Neurophysiologic monitoring of cerebral oxygen saturation at rest and at 5, 10, 15, and 20 minutes after intravenous administration of 15 mg/kg acetazolamide. Arterial blood was taken from a radial artery catheter in septic patients to perform blood gas analysis. Placement of arterial catheters in healthy control was considered unethical. F	Yes F	Complete follow up – all subjects accounted for. F	Fair

Note. Good quality: 3 or 4 stars (F) in selection domain AND 1 or 2 stars in comparability domain AND 2 or 3 stars in outcome domain; Fair quality: 2 stars in selection domain AND 1 or 2 stars in comparability domain AND 2 or 3 stars in outcome/exposure domain; Poor quality: 0 or 1 star in selection domain OR 0 stars in comparability domain OR 0 or 1 stars in outcome/exposure domain. Comparability: A) Controls for co-morbidities and/or other factors during recruitment B) Controls for confounding factors during statistical analysis

National Institutes of Health Quality Assessment Tool for Case Series

Rosenblatt et al. (2019) – *Quality Score: Good*

Criteria	Yes	No	Other (CD, NR, NA)
**Was the study question or objective clearly stated?**	X		
**Was the study population clearly and fully described, including a case definition?**	X		
**Were the cases consecutive?**		X	
**Were the subjects comparable?**	X		
**Was the intervention clearly described?**	X		
**Were the outcome measures clearly defined, valid, reliable, and implemented consistently across all study participants?**	X		
**Was the length of follow-up adequate?**	X		
**Were the statistical methods well-described?**	X		
**Were the results well-described?**	X		
